# Superior reproducibility and femoral tunnel angulation with hybrid transtibial vs. anteromedial portal techniques in ACL reconstruction: a retrospective case-control study

**DOI:** 10.3389/fsurg.2025.1594008

**Published:** 2025-11-04

**Authors:** Jiatong Li, Jie Wang, Qingjun Yang, Xiancheng Huang, Yong Luo, Sufen Ye, Haochi Lun, Tian You

**Affiliations:** 1 The Fifth Affiliated Hospital of Zunyi Medical University, Zhuhai, China; 2 Peking University Shenzhen Hospital, Shenzhen, Guangdong, China; 3 Tianjin Hospital, Tianjin, China; 4 Shandong Second Medical University, Weifang, Shandong, China; 5 Huizhou First People's Hospital, Huizhou, Guangdong, China; 6 Clinical Medical College, Shantou University, Shantou, Guangdong, China

**Keywords:** hybrid transtibial, anteromedial portal, anterior cruciate ligament, bone tunnel, anatomy

## Abstract

**Introduction:**

There are two traditional methods of femoral tunnel drilling during anterior cruciate ligament reconstruction (ACLR), transtibial (TT) or anteromedial portal (AM). However, both these approaches have specific disadvantages. Recently, a new technique combining the advantages of both approaches while avoiding their drawbacks has been developed, hybrid transtibial (HTT). The aim of the present study was to compare the radiology of the HTT and AM techniques in patients undergoing ACLR.

**Methods:**

We retrospectively analysed the three-dimensional computed tomography data of 31 patients who underwent ACLR (HTT and AM) at our institution between 29 October 2019 and 6 February 2023. The distance between the actual bone tunnel position and the standard anatomical location was measured in both the anterior–posterior and superior–inferior directions and expressed as a percentage. The spatial graft bending angle between the tibial and femoral tunnels was evaluated using Mimics software.

**Results:**

Thirty-one patients were included in the study: 12 and 19 in the AM and HTT groups, respectively. Compared with the AM group (9.71 ± 3.96, 9.37 ± 3.41), the HTT group had significantly smaller percentage distances t% (4.54 ± 2.76) in the anterior and posterior directions, and percentage h% (6.84 ± 2.66) in the upward and downward directions (*P* = 0.0002, *P* = 0.0281). The bending angles of the grafts in the AM and HTT groups were 103.79 ± 8.49 and 115.22 ± 9.72, respectively (*P* = 0.002), and the AM composition angle was more pronounced.

**Conclusions:**

The HTT technique exhibits superior repeatability in femoral tunnel drilling compared to the AM technique, facilitating more consistent achievement of the optimal graft bone tunnel position. Moreover, the graft bending angle observed with the AM technique is more pronounced than with HTT, which likely increases the forces exerted on the graft at the shallow edge of the tunnel aperture.

## 1 Introduction

ACL rupture is one of the most common sports injuries worldwide. The ACL is an important and stable structure of the knee joint that mainly limits the anterior translation and internal rotation of the tibia. Hence, patients with ACL injuries can experience instability in the anterior translation and rotation of the tibia (1).

ACLR surgery is commonly performed to treat ACL ruptures. For ACLR surgery, there are two traditional methods of femoral tunnel drilling: (1) establishing a femoral tunnel through the TT; or (2) placing a femoral tunnel through the AM approach (2). With regards to the two drilling methods, some scholars believe that AM technique can achieve more anatomical ACLR, which can improve the rotational stability and kinematics of the knee joint to obtain better clinical results (3–7). However, while obtaining anatomical femoral sites, this method faces technical challenges, and the resulting bone tunnels are not ideal, sometimes leading to excessively short tunnels or posterior wall blowouts, which can compromise tunnel integrity and negatively affect surgical outcomes (8, 9). This is in part due to the fact that the knee joint must undergo excessive flexion with this technique, making it difficult to obtain a familiar and consistent view of the lateral wall of the incision during surgery (10). The TT technique is surgically straightforward; however, it often results in suboptimal anatomical positioning of the femoral-side graft, which can compromise knee kinematics and lead to graft failure or reduced stability (7). Recently, a new technique, HTT, has been developed that combines the advantages of both the TT and AM approaches to create anatomical femoral tunnel sites without the need for excessive knee flexion, while maintaining optimal tunnel length, integrity, and angulation (11).

An important goal of ACLR is to approximate the reconstructed anatomical structure, and the location of the bone tunnels is crucial for achieving approximate anatomical reconstruction and postoperative efficacy (12–15). Although there are many reasons for ACLR failure, bone tunnel-related complications are among the most common (16). Literature indicates that improper positioning of the tunnel can increase the mechanical stress between the graft and tunnel (17). An incorrect bone tunnel position is associated with poor patient-reported results, knee joint loosening, transplant failure, and revision (18–20). Although the HTT technique can create anatomically compliant femoral tunnel sites compared with the AM technique, previous studies have primarily focused on positional accuracy without addressing the reproducibility of tunnel placement. In this study, we found that the HTT technique demonstrates better repeatability than the AM technique, as indicated by smaller deviations from the standard anatomical position, which may lead to more consistent surgical outcomes.

Moreover, prior studies typically assessed graft angulation in only the coronal and sagittal planes, which provides a limited two-dimensional perspective. In contrast, we developed a novel, convenient, and intuitive three-dimensional (3D) measurement method using Mimics software to directly evaluate the spatial bending angle of the graft, offering a more accurate and realistic reflection of graft orientation and biomechanics.

Hence, the aim of this study was to determine whether there is a difference in repeatability between the AM and HTT techniques, to provide orthopaedic surgeons with more reliable guidance when selecting femoral tunnel drilling methods during ACL reconstruction.

## 2 Materials & methods

### 2.1 Patients

The study was approved by an appropriate institution and all methods were performed in accordance with the relevant guidelines and regulations. We retrospectively analysed the 3D-computed tomography (CT) data of 31 patients who underwent ACLR surgery using the AM (*n* = 12) and HTT techniques (*n* = 19) from 29 October 2019 to 6 February 2023. All patients included in the study underwent 3D-CT examination on the first day after surgery, with all imaging performed with the knee positioned at 0°. Inclusion criteria included (1) a complete transphyseal reconstruction using any graft source; (2) use of a HTT, or AM femoral drilling technique; and (3) a clearly visible physis or physeal scar. Exclusion criteria included (1) revision ACL reconstruction, (2) multiligament knee reconstruction, (3) any other femoral drilling techniques except those mentioned in the inclusion criteria. All patients were operated on by two orthopaedic doctors at the same institution, each using the AM and HTT techniques. At the time of surgery, the tibial tunnel is created with a starting point 30 mm distal to the tibia.

The starting point is located 30 mm distal and 15 mm medial to the joint line. The tibial tunnel is created 15 mm medial to the inner edge of the tibial tuberosity. A stiff guide wire is drilled from this starting point into the center through the anterior cruciate ligament of the tibia, then use a cannulated straight stiff reamer. The femoral tunnel is then created using one of the following techniques:

1. HTT technique: The method of preparing the tibial tunnel at the beginning is similar to Jennings et al. (21). Unlike the primary HTT, in this study, a 2.0 mm K-wire was placed inside the tibia tunnel to reach the nearly isometric femoral point, in order to ensure graft tension stability during knee motion. Because the 2.0 mm Kirschner wire was thin and flexible, it allowed for a greater adjustment space. Usually, the 2.0 mm Kirschner wire allows for the tip to reach the edge of the femoral site, but can not reach the center. Thus, a vascular forceps was placed through the AM portal. Then, the K-wire was clamped, pushed into the femoral site, and tightened by a hammer. Although we did not have the Pathfinder ACL Guide or flexible Guide Pin, we can still achieve HTT technique through this method. After confirmation of correct placement, the femoral tunnel was reamed with a 7.5 mm drill from the outside to the inside of the joint and the LARS device was fixed at last. To standardize surgical conditions and precisely define key concepts (11): (1) “Nearly isometric femoral point” in the HTT technique was operationally defined as the position where graft tension variation remained ≤2 mm during passive knee motion (0°–120° flexion), identified by intraoperative tension testing with a calibrated force probe. This point ensures biomechanical stability while approximating the native ACL's functional behavior. (2) Knee flexion angles were strictly controlled: HTT group: 90° flexion during K-wire placement. AM group: 120° flexion during drilling.(3) Verification protocol: Fluoroscopic imaging (±5° tolerance) and tension measurements were documented for all cases.
2. Am technique: Similar to HTT technique, AM technique is also positioned at the isometric femoral point. However, in contrast to HTT, the drilling of the femoral tunnel is accomplished via the AM portal.

To standardize intraoperative variables across both techniques, all procedures adhered to strict protocols: (1) Flexion angles were controlled using a calibrated goniometer—90° for femoral drilling in the HTT group and 120° in the AM group, with graft fixation at 20° for both; (2) Positioning consistency was ensured via a knee holder (Smith & Nephew catalog #KN-2023) and maintained arthroscopic fluid pressure at 50 mmHg; (3) Verification of angles and tunnel placement was performed intraoperatively with C-arm fluoroscopy, confirming adherence within ±5° tolerance; and (4) Surgeon expertise was standardized, as all operations were conducted by two senior orthopedic surgeons, each with >4 years of specialized ACL reconstruction experience, following identical institutional protocols. This comprehensive approach minimized technical variability and ensured fair technique comparison.

### 2.2 Measurements

**Figure 1:**
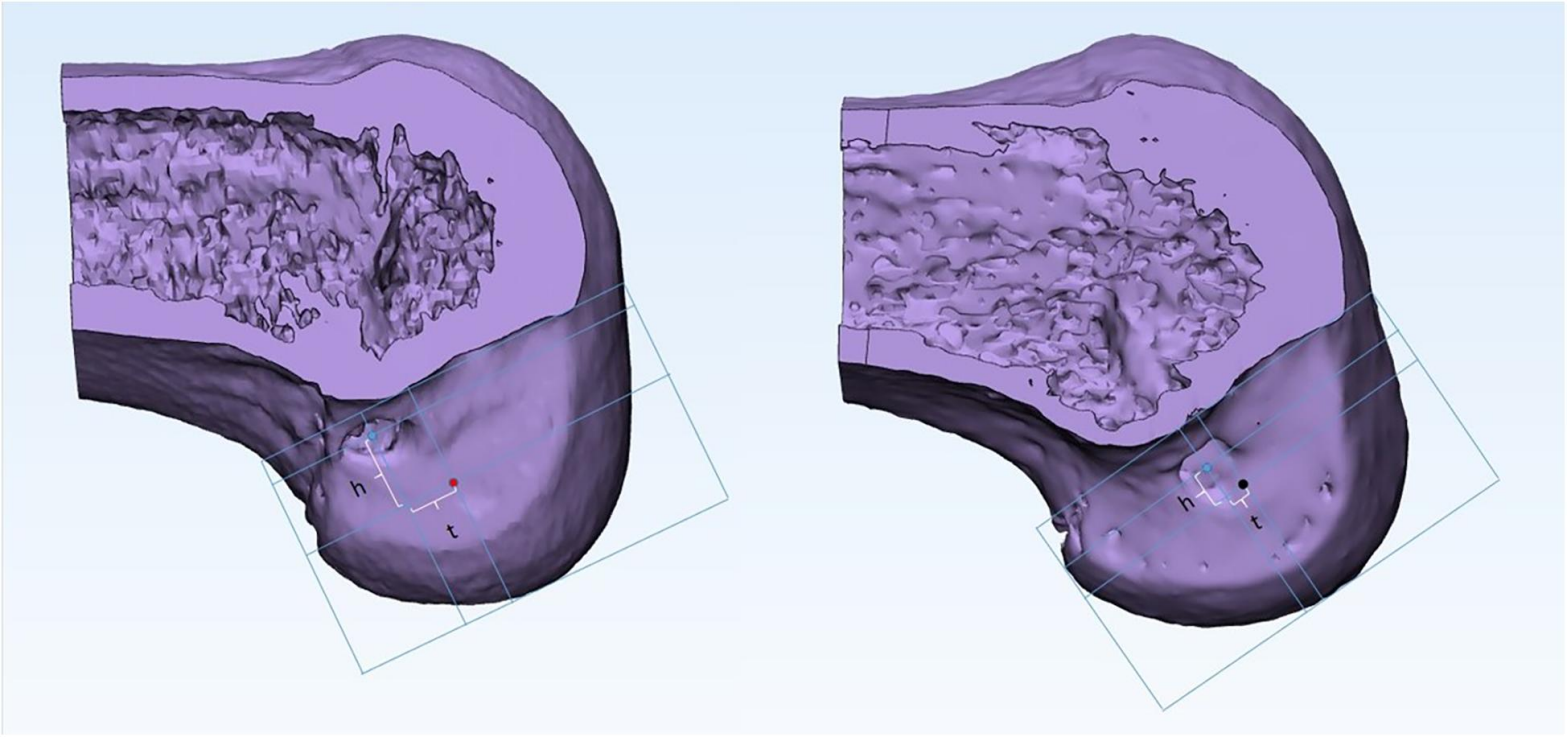
Schematic diagram representing distance between midpoint of the actual bone tunnel and standard site (left: AM; right: HTT). The blue dot represents the midpoint of the actual bone tunnel; the red dot represents the standard bone tunnel site of anteromedial portal; black dots are the standard bone tunnel sites for hybrid transtibial. The morphometric reference framework defined H (superior-inferior height) and T (anterior-posterior depth) as vertical and horizontal anatomical boundaries of the lateral femoral notch. Deviation was quantified by h (distance along H-axis) and t (distance along T-axis) between the actual tunnel midpoint and standard site. Percentage normalization (h%, t%) enabled size-invariant comparisons across patients.

**Figure 2:**
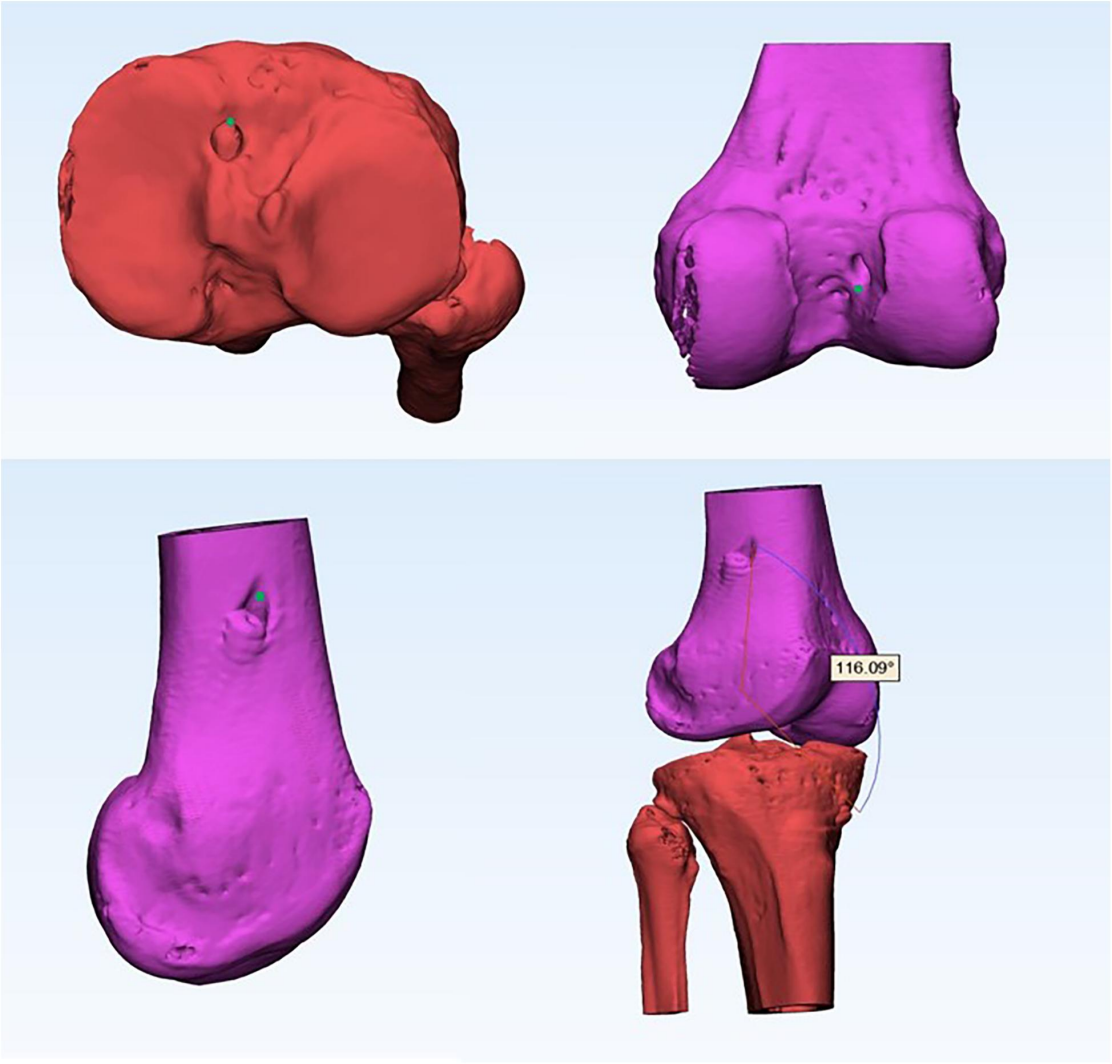
Schematic diagram measuring the curvature of the ACLR graft in a 3D view. The green dot represents the position where the angle is measured. ACLR, anterior cruciate ligament reconstruction; 3D, three-dimensional.

Using Mimics research software (version 19.0; Materialise, Inc., Leuven, Belgium) for 3D reconstruction of CT data (precise observation of bone tunnels in 3D views (22), the femur was divided into two parts along the highest point of the intercondylar ridge, and the lateral part was measured. Based on previous research, we defined the standard site as approximately 40% of the proximal-to-distal distance of the lateral notch, centered between the lateral intercondylar ridge and the posterior articular margin (23). According to the Bernard Quadrant method, the femur was scribed and the height(h) between the midpoint of the actual bone tunnel and the standard site in the anterior and posterior directions, as well as the depth(t) in the upper and lower directions (24) (Figure 1) were measured. These measurements were then converted into percentages using the following formulas: h% = h/H and t% = t/T, where H represents the total height (superior-inferior length) of the lateral notch and T represents the total depth (anterior-posterior length) of the lateral notch. However, because the tibial bone tunnel was located based on the residual ACL, we did not measure or analyse the tibial side. The measurement of the curvature of the ACL graft is shown in Figure 2. In brief, CT data were initially reconstructed in 3D using Mimics software. The graft position was defined by the apertures of the tibial tunnel, distal femoral tunnel, and proximal femoral tunnel. Subsequently, the graft bending angle was measured accurately using the software. All surgeries in this study were performed by two orthopedic surgeons, each with over four years of experience in anterior cruciate ligament reconstruction. Both surgeons were highly proficient in both the AM and HTT techniques and adhered to standardized surgical protocols at the same institution. All measurements were conducted by two experienced clinicians were blinded to the drilling method, with reference to (25) for standard loci, and the average of the measurement values was used for statistical analysis. Two observers each performed measurements three times, and the average value was taken as the final result to minimize inter-observer variability. Inter- and intra-observer reliability was assessed using intraclass correlation coefficients(ICCs). Two blinded clinicians performed measurements twice at 2-week intervals. ICCs(model 2,1) were 0.91(95% CI: 0.85–0.95) for t% distance and 0.89(95% CI: 0.82–0.93) for graft bending angles, indicating excellent agreement.

### 2.3 Statistical analysis

All eligible patients at our institution were included in the study; therefore, there was no prior calculation of the sample size. Nevertheless, we compared our results with those of relevant studies to evaluate the effect size of the study (26). The K-S test was used to test the normality of the data, independent sample *t*-tests were used for age, h, t, and angle, and the Fisher's exact probability test was used for classification of the data side and sex. *P*-value less than 0.05 indicates statistical significance. A *post hoc* power analysis was performed for the primary outcome (anterior-posterior tunnel distance, t%) using G*Power 3.1. Based on an assumed effect size of Cohen's d = 1.52 (α = 0.05, power = 0.99), the estimated sample size required was 16 patients per group (total *N* = 32) for an independent t-test. Although the AM group included 12 patients (slightly below the target), the actual effect size (d ≈ 1.58) yielded a statistical power >99%, confirming result robustness. Surgical side was analyzed as a binary covariate (0: left knee, 1: right knee) to quantify laterality effects. Multivariate linear regression models were constructed to control for covariates (age, sex, surgical side). Surgical side was encoded as a binary variable (0: left knee, 1: right knee) per orthopedics research standards.

## 3 Results

Thirty-one patients who underwent ACLR surgery using the AM and HTT techniques at [Masked for review] 29 October 2019 to 6 February 2023 were included in the present study. The patients were divided into two groups: AM (*n* = 12) and HTT (*n* = 19). The average age ± standard deviation (SD) of the AM and HTT groups were 31.58 ± 6.58 and 30.05 ± 8.57, respectively. The number of surgical sides (left/right) was 2/10 and 9/10, respectively, for the AM and HTT groups. The sex distribution (male/female) was 11/1 and 16/3, respectively, for the AM and HTT groups. There were no statistically significant differences in age, surgical side, and sex between the two groups (*P* = 0.602, 0.128, and 1.000, respectively). The two groups were analysed using independent sample t-tests for age and Fisher's exact probability test for surgical side and sex (Table 1).

**Table 1:** Patient demographic and characteristics.

Characteristics	AM (*n* = 12)	HTT (*n* = 19)	*P* Value
Age, mean ± SD, y	31.58 ± 6.58	30.05 ± 8.57	0.602
Side, left/right, *n*	2/10	9/10	0.128
Sex, male/female, *n*	11/1	16/3	1.000

AM, anteromedial; HTT, hybrid transtibial; SD, standard deviation; *T*-test using independent samples for age; Fisher's exact probability test was used for gender and surgical side (left/right).

The means ± SD of percentage distance t% in the anterior and posterior directions of the AM and HTT groups were 9.71 ± 3.96 and 4.54 ± 2.76, respectively, and the means ± SD of percentage distance h% in the upper and lower directions were 9.37 ± 3.41 and 6.84 ± 2.66, respectively. The mean gradient angles ± SD were 103.79 ± 8.49 and 115.22 ± 9.72, respectively, in the AM and HTT groups. There were statistically significant differences between the two groups in terms of distance t, distance h, and grade bonding angles (*P* = 0.0002, 0.0281, and 0.002, respectively) (Table 2). In the multivariate linear regression analysis adjusted for age, sex, and surgical side (right vs. left), surgical technique (HTT vs. AM) emerged as the sole significant predictor for both t% distance and graft bending angle. Specifically, HTT was associated with a 5.17% reduction in t% distance [β = −5.17, 95% CI (−7.24, −3.10), *P* < 0.001] and an 11.43° increase in graft bending angle [β = 11.43, 95% CI (4.82, 18.04), *P* = 0.001] compared to AM. None of the covariates—age (β = 0.04, *P* = 0.78), sex (β = 0.12, *P* = 0.65), or surgical side (β = 0.08, *P* = 0.41)—showed statistically significant associations with either outcome. Notably, the absence of a significant effect for surgical laterality (*P* > 0.05) provides evidence supporting anatomical symmetry in the outcomes of anterior cruciate ligament (ACL) reconstruction. Regression analysis adjusting for demographic and anatomic covariates confirmed technique-dependent differences (Table 3).

**Table 2:** Tunnel and aperture characteristics.

Characteristics	AM (*n* = 12)	HTT (*n* = 19)	*P* Value
t%, mean ± SD	9.71 ± 3.96	4.54 ± 2.76	**0** **.** **0002**
h%, mean ± SD	9.37 ± 3.41	6.84 ± 2.66	**0**.**0281**
Mean graft bending angle ± SD, deg	103.79 ± 8.49	115.22 ± 9.72	**0**.**002**

AM, anteromedial; HTT, hybrid transtibial; SD, standard deviation; t = distance in the anterior and posterior directions; h = distance in the upward and downward directions; T, H (Figure 1); t% = t/T; h% = h/H. Using *t*-tests with independent samples, bold indicates statistical significance (*P* < 0.05).

**Table 3:** Multivariate linear regression analysis of factors influencing tunnel positioning and graft angulation.

Variable	t% distance β (95% CI)	Graft bending angle *P* value
Technique (HTT vs. AM)	−5.17 (−7.24, −3.10)	**<0** **.** **001**
Age	0.04 (−0.25, 0.33)	0.78
Sex (male vs. female)	0.12 (−0.41, 0.65)	0.65
Side (right vs. left)	0.08 (−0.15, 0.31)	0.41

AM, anteromedial; HTT, hybrid transtibial; β, standardized regression coefficient; CI, confidence interval. Bolded *P* values indicate statistical significance (*P* < 0.05).

## 4 Discussion

The main finding of this study was that the bone tunnel drilled using HTT technique was closer to the standard site (whether in the anterior and posterior or upward and downward directions) than that drilled using AM technique. This is crucial information for orthopaedic doctors that can inform their choice of drilling method, as it allows them to drill bone tunnels that are more in line with standard sites. The femoral tunnel is crucial for surgical efficacy. In terms of better function, doctors have attempted to reproduce the anatomy as closely as possible by drilling the femoral tunnel in the center of the native ACL insertion (27, 28). However, in recent years, IEDAL or near Isometric reconstruction have become more highly regarded by surgeons (29). The theory refers to tunnel placement such that the distance between the tibial and femoral tunnel apertures remains constant as the knee moves from extension to flexion. Prospective clinical studies have shown that differences in tunnel location can significantly affect the graft healing and the clinical outcome scores (30). In the present study, the difference between the two groups in the anterior and posterior directions was more significant than the difference in the upper and lower directions, which may be because orthopaedic surgeons use the intercondylar spine as a reference in the upper and lower directions when using arthroscopy for ACLR surgery, which allows better evaluation of the position of the drilling hole. Therefore, orthopaedic surgeons may need to pay more attention to the position of the tunnel in the anterior and posterior directions during surgery.

Another finding of this study is that 3D software can be used to measure the bending angle of the graft easily and quickly. Herein, we found that the spatial angulation of the grafts in the AM group was significantly greater than that of the HTT group (*P* < 0.05). This is consistent with previous research showing that increasing the bending angle of the graft can also increase the force on the edge of the tunnel opening, which may be detrimental to the prognosis of the graft (31).

The advantage of this study is that it measures the 3D angle of the graft rather than the two-dimensional angle, which makes the measurements more accurate, and moreover, the measurement method is simple and easy to perform (11). Moreover, the position of the bone tunnel also affects the graft bending angle. During ACLR, as the knee flexion angle increases, the femoral tunnel aperture moves anteriorly and distally, resulting in a smaller graft bending angle (32). A study using computer simulations reached a similar conclusion, indicating that placing the femoral aperture more proximally can reduce the graft bending angle without altering the anatomical position of the femoral and tibial tunnels (33). Therefore, during surgery, attention should be paid to maintaining an appropriate knee flexion angle and to the positioning of the femoral tunnel aperture.

Previous studies have demonstrated that the HTT technique can achieve anatomically compliant femoral tunnel positioning compared to the AM technique (11, 34). However, these studies primarily focused on positional accuracy without addressing the reproducibility of tunnel placement.In contrast, our study highlights two key innovations. First, we quantitatively evaluated the repeatability of femoral tunnel positioning, revealing that the HTT technique offers superior consistency compared to the AM technique. This suggests that HTT may provide more predictable and reliable surgical outcomes. Second, we developed a novel, convenient, and intuitive three-dimensional (3D) measurement method using Mimics software to directly assess graft curvature. Unlike prior assessments limited to coronal and sagittal planes, our 3D approach offers a more comprehensive and realistic evaluation of graft angulation and biomechanics, which may have implications for graft maturation and clinical results. By emphasizing these aspects, our work advances the current understanding of ACL reconstruction techniques and provides valuable reference data for clinical decision-making.

TT technique is widely used in ACLR (8). It has advantages, such as low technical requirements, good cosmetic properties, shorter surgical time, less surgical pain and complications, and low risk of bone tunnel bursts (35–40). However, TT technique also has many shortcomings, such as poor anatomical quality of femoral side grafts, mismatched length of graft tunnels, enlarged bone tunnels, inability to locate femoral tunnels separately, rotational instability caused by vertical grafts, impact with the posterior cruciate ligament, possible invasion of the posterior cortex, graft displacement, and increased graft stress (36, 37, 40–44). The AM and TT technologies both have advantages and disadvantages. AM technique can obtain more anatomical transplant sites, and the tunnels on the tibial and femoral sides can be placed independently, with screws placed in parallel, allowing for flexible single- or double-bundle reconstruction, preserving ACL stumps, and providing faster return to activity compared to TT, with better stability in both anteroposterior and rotational aspects (37, 39, 41, 44). However, the AM technique has several drawbacks. A limited field of vision and excessive flexion may cause tunnel enlargement (4, 30, 42, 45). Compared to TT technique, the anteromedial (AM) technique requires a higher level of surgical skill from the operator. Reduced visualization during the anteromedial (AM) technique implies that tunnel positioning relies more heavily on the surgeon's experience, which may contribute to its lower reproducibility (46).

Given the many advantages and disadvantages of the TT and AM technologies, a new technique, HTT, has been developed that combines the advantages of these technologies while avoiding their drawbacks. Finite element analysis showed that HTT technique is a true hybrid of AM and TT techniques, which avoids excessive knee flexion and maintains the direction and length of the femoral canal, similar to TT technique, but also achieves anatomical localisation of AM grafts (11, 21, 25). It is generally accepted that HTT is an ideal choice for adolescent ACLR. Compared with AM, HTT causes less damage to the epiphysis and is more conducive to growth and development (26).

This study had some limitations. This was purely an imaging study, and further research is required to determine whether there is a difference in the reproducibility of HTT and AM drilling for clinical efficacy. In addition, the isometric point positioning method was employed during the surgical procedure to ensure stable graft tension throughout knee joint motion; however, this approach may influence the positioning of the bone tunnels. Future studies should take this factor into consideration. This imaging-focused study lacks clinical outcome correlation (e.g., Lysholm scores), limiting direct applicability. Future work should integrate long-term follow-up to link tunnel reproducibility with functional recovery. Additionally, the isometric point targeting may influence anatomical positioning; prospective designs should address this.

Although the *post hoc* power analysis confirmed that the primary results had sufficient statistical power (>99%), the relatively small sample size of the AM group (*n* = 12) suggests that caution should be exercised when extrapolating these findings. Future large-scale prospective studies are necessary to further validate the conclusions of this research. In addition, the null effects of surgical laterality (*P* > 0.05) support anatomic symmetry in ACL reconstruction outcomes.

## 5 Conclusion

The findings of this imaging study indicate that HTT technique outperforms AM technique in terms of drilling repeatability. It is more beneficial for beginners and artificial ligament reconstruction. Moreover, the angularity of the AM technique graft space was greater than that of the HTT technique space.

## Data Availability

The datasets are available from the corresponding author on reasonable request.
